# From Yufeining to Kaempferol: Multi-Target Anti-Inflammatory, Antioxidant, and Anti-Apoptotic Mechanisms Targeting the STAT3–TP53–IL1B Signaling Network in COPD Therapy

**DOI:** 10.3390/molecules31101674

**Published:** 2026-05-15

**Authors:** Xiubo Li, Jiacheng Shi, Liyuan Pang, Kailin Zhong, Fan Bu, Sumreena Mansoor, Kan He

**Affiliations:** 1 Department of Pharmacology, College of Basic Medical Sciences, Jilin University, Changchun 130021, China; lixb9922@mails.jlu.edu.cn (X.L.); shijc25@mails.jlu.edu.cn (J.S.); zhongkl9922@mails.jlu.edu.cn (K.Z.); bufan9922@mails.jlu.edu.cn (F.B.); 2 State Key Laboratory of Advanced Drug Formulations for Overcoming Delivery Barriers, School of Pharmaceutical Sciences, Shanghai 201203, China; 25111030066@m.fudan.edu.cn; 3 Department of Biochemistry, Shifa College of Medicine, Shifa Tameer-e-Millat University, Islamabad 44000, Pakistan; sumreena.scm@stmu.edu.pk

**Keywords:** COPD, Yufeining, kaempferol, inflammation, oxidative damage, apoptosis

## Abstract

Background: Chronic obstructive pulmonary disease (COPD) is a heterogeneous syn-drome which leads to irreversible and progressive airflow limitation. Yufeining (YFN) is a Chinese herbal formula employed for treating COPD; however, the key ingredients and mechanisms are not fully defined. Methods: The key bioactive compounds and their corresponding targets of YFN were recognized using the network pharmacology method. The CIBERSORT algorithm was employed to identify the immune infiltration profiles in COPD and their correlation with the principal targets. Through the COPD mouse model, the effects of kaempferol on inflammation, oxidative damage, and apoptosis were evaluated. The BEAS-2B cells treated with cigarette smoke extract (CSE) were used to assess the protective effects of kaempferol against inflammation, oxidative stress, and apoptosis. Using flow cytometry, the anti-apoptosis effects of kaempferol were analyzed. Transcriptomic Analysis was performed to investigate the transcriptional changes in CSE-induced BEAS-2B cells. Results: Kaempferol is a key compound of YFN, and STAT3, TP53, and IL1B are predicted to be the core targets of YFN for COPD treatment. Immune infiltration analysis revealed a significant correlation between STAT3-TP53-IL1B signaling network and inflammatory cell infiltration. Kaempferol alleviated inflammation, oxidative damage, and apoptosis in the COPD mouse model. In CSE-induced BEAS-2B cells, kaempferol inhibited the inflammatory response, oxidative damage, and apoptosis. The transcriptome sequencing revealed a total of 223 differentially expressed genes. After treating with kaempferol, the transcriptional levels of STAT3 significantly increased, while those of TP53 and IL1B significantly decreased. Conclusions: Kaempferol can exert therapeutic effects against COPD by inhibiting inflammatory response and oxidative damage, and reducing cell apoptosis. Furthermore, this study indicated the therapeutic mechanism of YFN in COPD involves potentially targeting the STAT3-TP53-IL1B signaling network through kaempferol.

## 1. Introduction

COPD is a heterogeneous respiratory syndrome featuring persistent and progressive airflow limitation [1]. The primary factor leading to COPD is inhaling noxious particles, including cigarette smoke and pollutants. Additionally, COPD can also be caused by genetic, infectious, and social factors [2,3]. According to research, COPD is a major cause of morbidity and mortality in the world, and it is estimated that the number of COPD patients will approach 600 million globally by 2050, imposing a substantial financial strain on patients and society [4,5]. In current clinical practice, to relieve dyspnea and exercise intolerance and to prevent acute exacerbations, bronchodilators and glucocorticoids are used for COPD pharmacological management [6]. However, no current therapeutic intervention can effectively stop the progression of the disease [7].

Traditional Chinese medicines (TCM) possess significant multi-target properties, exhibiting unique advantages in the treatment of COPD [8]. YFN is a traditional Chinese herbal formula which primarily composed of 15 Chinese medical herbs, comprising Radix Codonopsis (DS), Radix Ledebouriellae (FF), Fructus Schisandrae (WWZ), Fructus Corni (SZY), Juglandis (HTR), Rhizoma Polygonati (HJ), Semen Cuscutae (TSZ), Radix Morindae Officinalis (BJT), Fructus Trichosanthis (GL), Rhizoma Pinelliae (FBX), Bulbus Fritillariae Thunbergii (ZBM), Rhizoma Atractylodis Macrocephalae (BZ), Radix Salviae Miltiorrhizae (DANS), Semen Persicae (TR), and Radix Astragali (HQ). YFN has achieved a clinical success in reducing coughing, expectoration, and chest tightness. Clinical data suggest that YFN has positive effects in treating stable COPD, including improvement of respiratory function and relief of airway inflammation. Related studies indicate that YFN may treat COPD through mechanisms involving the reduction of sputum total cell count, absolute neutrophil count, and IL-8 concentration [9,10]. The primary bioactive compounds of YFN and their therapeutic mechanisms, however, are not fully defined.

Kaempferol is a flavonoid compound found in various natural herbs and is recognized to exhibit anti-inflammatory and anti-apoptotic properties [11]. Evidence suggests that kaempferol possesses therapeutic potential for a range of chronic inflammatory conditions. For example, kaempferol has been reported to slow the progression of chronic kidney disease by suppressing the Piezo1/HIF-1a/ROS/NLRP3 signaling pathway [12]. Previous studies also demonstrate that kaempferol can attenuate idiopathic pulmonary fibrosis in experimental models through alleviating inflammatory infiltration and extracellular matrix deposition [13]. However, the therapeutic potential of kaempferol in COPD and its underlying mechanisms remain unclear.

Network pharmacology, a systematic method based on topological network analysis and virtual prediction, has unique advantages in multi-compound and multi-target analysis [14,15,16]. Through constructing and analyzing the network pharmacology diagram, the interactions between bioactive compounds and potential targets can be explored, and the disease pathways regulated by drug compounds can be clarified [17,18]. In this study, network pharmacology was utilized to identify the bioactive compounds of YFN and their corresponding targets. Subsequently, we employed molecular docking technology and molecular dynamics (MD) simulation to validate the interaction between drugs and target proteins. CIBERSORT analysis was applied to characterize the association of core targets with immune infiltration in COPD. Finally, both in vitro and in vivo experiments, together with transcriptomic analysis, were performed to investigate the mechanistic basis of the therapeutic effects of the main compounds against COPD. This comprehensive strategy helped clarify the underlying mechanisms of YFN for COPD therapy.

## 2. Results

### 2.1. Identification of Active Ingredients and Targets in YFN

By utilizing OB ≥ 30 and DL ≥ 0.18 as criteria in the TCMSP database, 21 bioactive ingredients of DS, 20 bioactive ingredients of HQ, 7 bioactive ingredients of BZ, 18 bioactive ingredients of FF, 12 bioactive ingredients of HJ, 20 bioactive ingredients of SZY, 21 bioactive ingredients of WWZ, 4 bioactive ingredients of HTR, 11 bioactive ingredients of TSZ, 20 bioactive ingredients of BJT, 11 bioactive ingredients of GL, 13 bioactive ingredients of FBX, 7 bioactive ingredients of ZBM, 65 bioactive ingredients of DANS, and 23 bioactive ingredients of TR were collected. Kaempferol is a bioactive component shared by HQ and TSZ. The overlapping bioactive compounds are shown in Table 1. Following deduplication, 289 targets related to YFN were collected from the UniProt database.

### 2.2. Component–Target Network

**Figure 1:**
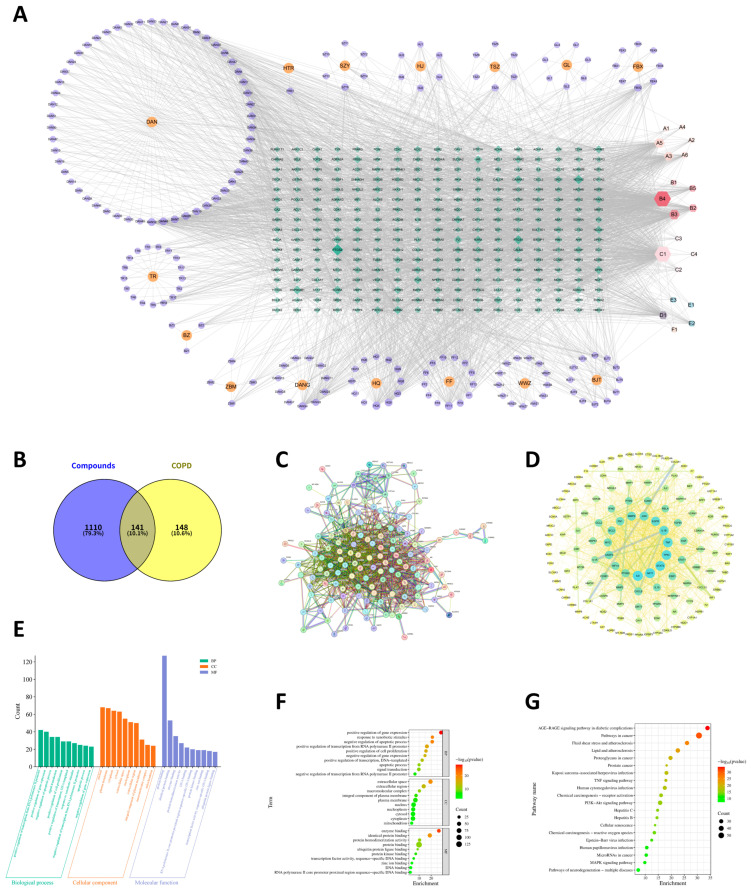
Network Pharmacology, GO, and KEGG analysis. (**A**) Compound–Target Network of YFN. (**B**) Venn diagram showing the overlapping targets between YFN and COPD. (**C**) The PPI network of YFN and COPD targets. (**D**) Core target genes of YFN and COPD. (**E**) Top enriched GO terms among the hub genes. (**F**) Bubble chart of the top 10 terms from GO enrichment analysis. (**G**) Bubble chart of the top 20 KEGG pathways of hub genes.

The interaction network of components and targets, comprising 15 herbs, 193 compounds, and 289 targets, was accomplished through Cytoscape 3.10.0 (Figure 1A). In the constructed network, compounds and targets are depicted as nodes, with the edges connecting them signifying their interrelationships. As shown in Table 2, through the analysis of the network diagram, quercetin (degree = 302), beta-sitosterol (degree = 296), and kaempferol (degree = 130) are the top three bioactive compounds ranked by degree values.

### 2.3. COPD-Related Targets Acquisition

The DrugBank database retrieved 104 disease target genes, the OMIM database retrieved 186 target genes, the therapeutic target database retrieved 56 target genes, and the GeneCards database retrieved 1087 target genes. After merging the datasets and removing duplicate entries, we finally identified 1251 genes relevant to COPD.

### 2.4. PPI Network

We first identified the intersection between drug-related and COPD-related target genes using the Venny database, which revealed 141 common genes (Figure 1B). The gene set was then imported into the STRING database to generate the network, with the organism filter set to “Homo sapiens” and the minimum required interaction confidence score set to 0.7 (Figure 1C). To further identify the core target genes, the protein–protein interaction information was further analyzed in Cytoscape 3.10.0. We calculated the parameters of each node with the Centiscape 2.2 plugin. Subsequently, key genes were selected according to degree values, betweenness centrality, and closeness centrality. Finally, STAT3, TP53, and IL1B were identified as the top-ranked genes for subsequent validation (Figure 1D).

### 2.5. GO and KEGG Analysis

The DAVID database was used for GO and KEGG pathway enrichment analysis of 141 common target genes, and the species was limited to “Homo sapiens”. The GO enrichment analysis identified a collection of 1060 GO items, including 840 terms related to BP, 81 terms related to CC, and 139 terms related to MF. Based on the number of enriched genes, false discovery rate (FDR), and *p*-value magnitude, the top 10 terms were selected (Figure 1E,F). The BP terms predominantly focus on positive regulation of transcription from RNA polymerase II promoter, positive regulation of gene expression, negative regulation of apoptotic process, signal transduction, and positive regulation of cell proliferation, etc. Highly enriched CC terms involve nucleus, plasma membrane, cytoplasm, cytosol, extracellular space, nucleoplasm, extracellular region, integral component of plasma membrane, macromolecular complex, and mitochondrion. Additionally, the MF terms with the highest enrichment are mainly linked to protein binding, identical protein binding, enzyme binding, protein homodimerization activity, DNA binding, RNA polymerase II core promoter proximal region sequence-specific DNA binding, protein kinase binding, zinc ion binding, transcription factor activity, sequence-specific DNA binding, and ubiquitin protein ligase binding.

From the KEGG enrichment analysis, 174 signaling pathways were retrieved. A bubble chart (Figure 1G) displays the ten potential pathways with the highest gene counts, which encompass pathways in cancer, the AGE-RAGE signaling pathway in diabetic complications, lipid and atherosclerosis, the PI3K-Akt signaling pathway, and fluid shear stress and atherosclerosis, etc.

### 2.6. Molecular Docking Results

**Figure 2:**
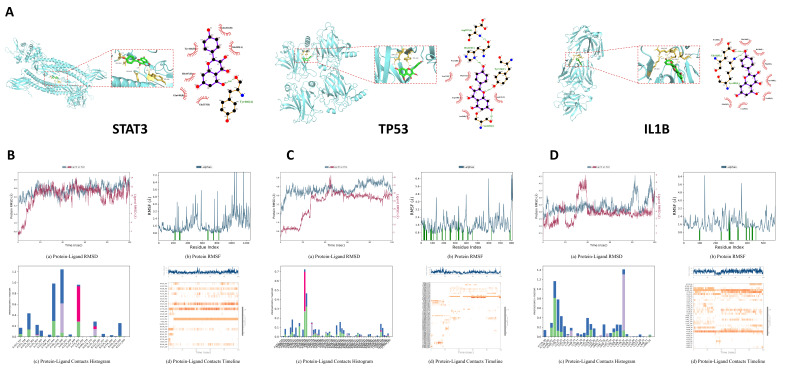
Molecular docking and MD simulation analysis. (**A**) Molecular docking of kaempferol with three key targets. (**B**) MD simulation results of kaempferol with STAT3. (**C**) MD simulation results of kaempferol with TP53. (**D**) MD simulation results of kaempferol with IL1B. Green vertical bars in the protein RMSF plot denote residues interacting with the ligand. Green, purple, magenta, and blue columns in the protein-ligand contacts histogram represent hydrogen bonds, hydrophobic contacts, ionic interactions, and water bridges, respectively.

Kaempferol and core targets (STAT3, TP53, and IL1B) identified from the network pharmacology analysis were screened for molecular docking validation. The resulting binding affinities for these compound–target complexes are shown in Table 3. The optimal image between the ligand and receptor is visualized (Figure 2A). The binding energies of kaempferol with STAT3, TP53, and IL1B were −7.686, −7.814, and −7.306 kcal/mol, respectively, suggesting a strong affinity between kaempferol and these targets.

### 2.7. MD Simulation Evaluation

#### 2.7.1. STAT3 with Kaempferol

The fluctuations of the protein skeletal atoms and ligand heavy atoms entered a more stable phase after about 20 ns. Residues that interact with the ligand exhibit less fluctuation compared to the rest of the protein regions. Protein residues like GLN361, GLU444, and LYS451 in chain A interact with the ligand by forming hydrogen bonds, and TYR446 in chain A and LYS282 in chain B contact with the ligand via hydrophobic interactions. The protein–ligand contacts timeline reveals that the bioactive compound has a stable interaction with GLU444, TYR446, and LYS451 in chain A. These analyses collectively confirm the structural stability of the STAT3–kaempferol complex (Figure 2B).

#### 2.7.2. TP53 with Kaempferol

The fluctuations of the protein skeletal atoms and ligand heavy atoms entered a more stable phase after about 50 ns. Residues that interact with the ligand exhibit less fluctuation compared to the rest of the protein regions. Protein residues like GLN136, LYS139, and THR140 in chain A interact with the ligand by forming hydrogen bonds, and HIS178 in chain A and PRO152 in chain B contact with the ligand via hydrophobic interactions. The protein–ligand contacts timeline indicates that the bioactive compound has a stable interaction with LYS139 in chain A. These analyses collectively confirm the structural stability of the TP53–kaempferol complex (Figure 2C).

#### 2.7.3. IL1B with Kaempferol

The fluctuations of the protein skeletal atoms and ligand heavy atoms entered a more stable phase after about 40 ns, but the ligand RMSD fluctuates largely from about 95 ns, indicating significant conformational changes. Residues that interact with the ligand exhibit less fluctuation compared to the rest of the protein regions. Protein residues like ASN101, ASP103 in chain H, and GLN81 in chain I interact with the ligand by forming hydrogen bonds, and LYS74 in chain I and TYR32 in chain L contact with the ligand via hydrophobic interactions. The protein–ligand contacts timeline indicates that the bioactive compound has a stable interaction with ASP103 in chain H and TYR32 in chain L. These analyses collectively confirm the structural stability of the IL1B–kaempferol complex (Figure 2D).

### 2.8. Identification of DEGs

**Figure 3:**
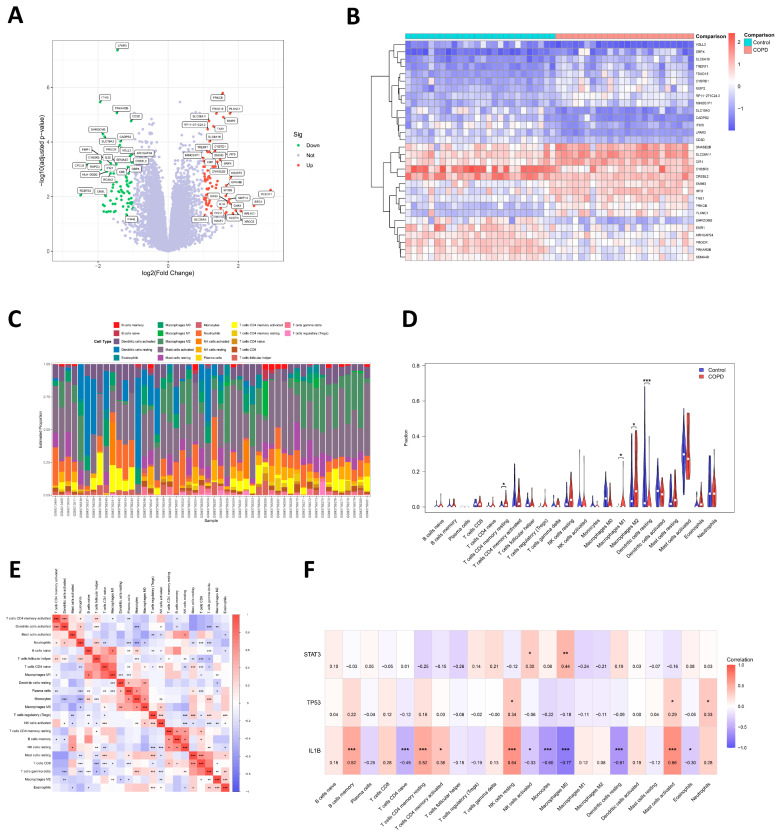
DEGs identification and immune infiltration characteristics of the GSE130928 and GSE8608 datasets. (**A**) Volcano plot of DEGs. (**B**) Heatmap showing the expression of the top 30 DEGs between COPD patients and healthy controls. (**C**) Composition of infiltrating immune cell types across samples. (**D**) Comparative analysis of immune infiltration across groups. (**E**) Heatmap presenting the correlation across the 22 types of immune cells. (**F**) Associations of STAT3, TP53, and IL1B with 22 immune cell types. * *p* < 0.05, ** *p* < 0.01, *** *p* < 0.001.

Differential gene expression analysis was conducted on 50 samples from datasets GSE130928 and GSE8608, comparing 24 COPD cases to 26 controls. Applying the “limma” package with a cut-off of |logFC| > 1 and an adjusted *p*-value < 0.05, a total of 147 DEGs were detected, comprising 72 upregulated and 75 downregulated genes (Figure 3A,B).

### 2.9. Immune-Infiltrating Analysis

To characterize the immune microenvironment in COPD, we applied the CIBERSORT algorithm to profile the landscape of immune cell infiltration. Figure 3C illustrates the composition of 22 immune cells in each sample, while Figure 3E presents interrelationships between different infiltrating immune cell types. The comparative analysis reveals that the levels of resting memory CD4+ T cells, M1 macrophages, and M2 macrophages were higher in the COPD patients relative to controls (Figure 3D). Next, we investigated the correlation between the top three core target genes and immune cells, and strong correlations were found. STAT3 had a positive correlation with activated NK cells and M0 macrophages. TP53 shows a positive correlation with resting NK cells, neutrophils, and activated mast cells. IL1B showed positive associations with activated mast cells, resting NK cells, memory B cells, resting memory CD4+ T cells, etc., yet negative associations with M0 macrophages, resting dendritic cells, monocytes, etc. (Figure 3F). Based on the analysis results, the elevation of the resting memory CD4+ T cells and M1 macrophage levels may contribute to COPD-related pathogenesis and progression. Through acting on STAT3, TP53, and IL1B, YFN may modulate the immune-infiltration cell types to exert its therapeutic effect on COPD.

### 2.10. Kaempferol Is Capable of Ameliorating Pathological Alterations in Lung Tissue and Reducing Collagen Deposition Around Airways in a Murine Model of COPD

**Figure 4:**
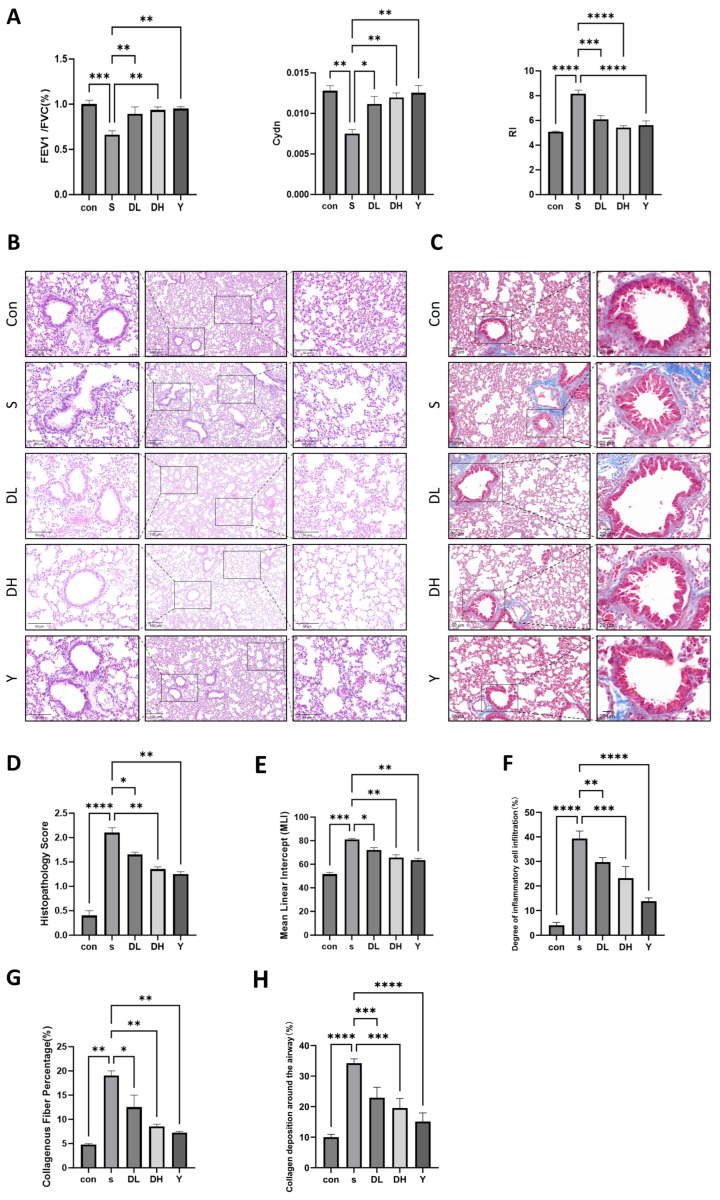
Effects of kaempferol on pulmonary function, lung pathology, and collagen deposition in mice with COPD. (**A**) Pulmonary function testing with FEV1/FVC (%), Cdyn, and RI used as indicators, respectively. (**B**) HE staining of the lung tissues of mice. HE staining and (**C**) Masson staining of lung tissues of mice. (**D**) Pathological evaluation of mouse lung tissues. (**E**) Mean linear intercept (MLI) analysis. (**F**) Degree of inflammatory cell infiltration. (**G**) The collagen fibrosis area ratio of lung tissues. (**H**) Extent of collagen deposition around airways. Scale bar 100 μm, 50 μm and 20 μm. Relative to the smoke group, * *p* < 0.05, ** *p* < 0.01, *** *p* < 0.001, **** *p* < 0.0001.

The effect of kaempferol on pulmonary function was evaluated in mouse models of COPD. Results showed that relative to controls, smoke-exposed mice exhibited markedly lower FEV1/FVC (%) and Cdyn, yet markedly higher RI, reflecting obstructive airway injury and successful establishment of cigarette smoke-induced COPD. Administration of different doses of kaempferol and dexamethasone both increased FEV1/FVC (%) and Cdyn, and decreased RI in mice (Figure 4A). Using HE staining, histological examination revealed no obvious pathological lesions in the lung tissues of the blank control group, with normal structural integrity and physiological function. In contrast, mice in the model group developed marked pathological changes in lung tissue (Figure 4B,D), characterized by inflammatory cell infiltration, alveolar wall destruction, and alveolar cavity enlargement. Intervention with different doses of kaempferol and dexamethasone significantly alleviated these pathological manifestations (Figure 4E,F). Masson staining for histopathological analysis of lung tissues from COPD mice showed that there was no obvious collagen fiber deposition or fibrous foci formation in the lung tissue of the control group. Conversely, extensive collagen fiber deposition was detected in the model group (Figure 4C), which confirmed that long-term cigarette smoke exposure induces the collagen fiber content in lung parenchymal tissues and collagen deposition around airways. After intervention with dexamethasone and different doses of kaempferol, all experimental groups showed a marked reduction in fibrotic changes compared with the model group (Figure 4G,H). Collectively, these findings demonstrated that kaempferol can ameliorate pathological alterations in lung tissue and reduce collagen deposition around airways induced by cigarette smoke exposure in mice.

### 2.11. Kaempferol Suppresses Inflammation, Reduces Oxidative Damage, and Inhibits Apoptosis in Lung Tissue of COPD Mouse Models

**Figure 5:**
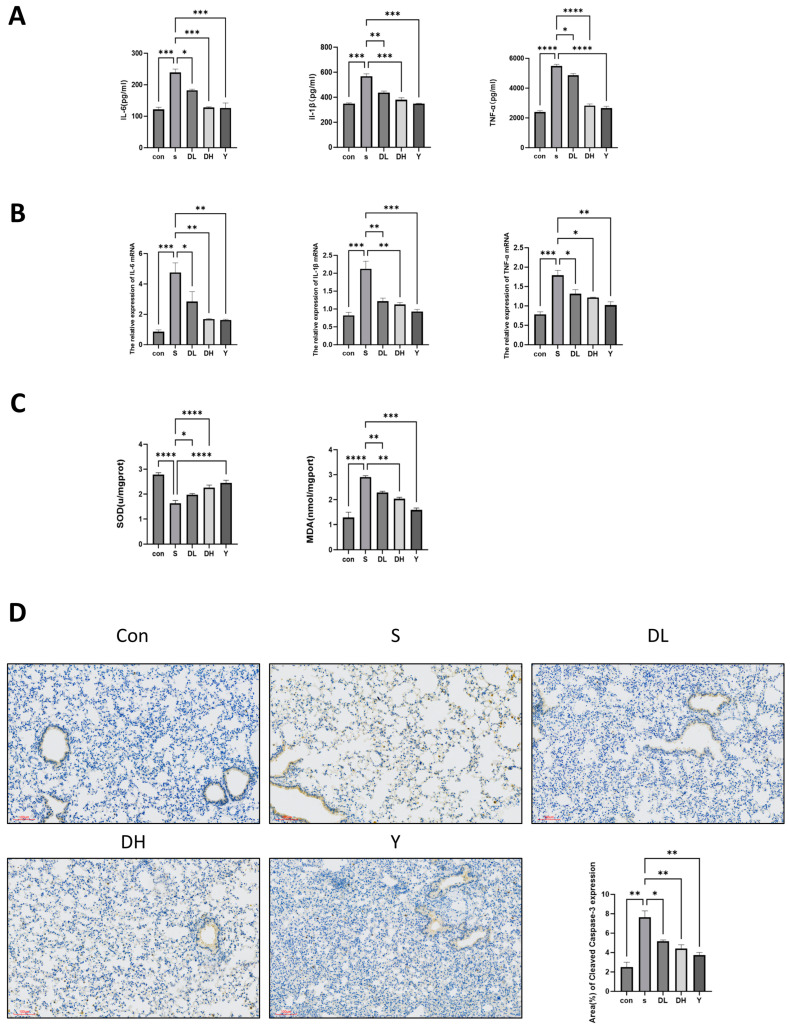
Kaempferol ameliorates inflammation, oxidative damage, and apoptosis in a mouse model of COPD. (**A**) ELISA-based detection of IL-6, IL-1β, and TNF-α. (**B**) RT-qPCR was used to detect IL-6, IL-1β, and TNF-α. (**C**) The contents of SOD and MDA in the lung tissues of each group of mice. (**D**) IHC detection of the positive expression of Cleaved caspase-3. * *p* < 0.05, ** *p* < 0.01, *** *p* < 0.001, **** *p* < 0.0001.

The levels of inflammatory factors in the lung tissues of the COPD model mice were determined by ELISA and RT-qPCR. Following cigarette smoke exposure, the levels of IL-6, IL-1β, and TNF-α in lung tissues of the model group were markedly elevated (Figure 5A). Relative to the model group, administration of kaempferol and dexamethasone both significantly reduced the concentrations of these inflammatory mediators in lung tissue, and the inhibitory effect of kaempferol was enhanced with increasing doses. Furthermore, compared with the control group, the mRNA abundance of IL-6, IL-1β, and TNF-α was considerably higher in the model group (Figure 5B). Conversely, treatment with both kaempferol and dexamethasone markedly alleviated the elevated mRNA transcript quantities for IL-6, IL-1β, and TNF-α, and the action of kaempferol showed a dose-dependent pattern. The antioxidant capacity of kaempferol in lung tissues of COPD mice was assessed by detecting the contents of SOD and MDA (Figure 5C). Relative to the control group, SOD content was decreased while MDA content was increased in the model group. Following kaempferol intervention, the content of SOD was elevated, and the content of MDA was reduced, suggesting that kaempferol may exert antioxidant effects and effectively mitigate cigarette smoke-induced pulmonary oxidative injury. In addition, IHC was employed to evaluate Cleaved caspase-3 expression in lung tissues of COPD mice following kaempferol treatment (Figure 5D). Versus the control group, the abundance of Cleaved caspase-3 isolated from lung tissues was significantly higher in the model group. Conversely, upon exposure to kaempferol and dexamethasone, the content of inflammation-related Cleaved caspase-3 in mouse lung tissues displayed a decreasing trend compared with the model group, demonstrating that kaempferol could inhibit apoptosis to a certain extent. These results indicated that kaempferol can alleviate the inflammatory response, oxidative damage, and apoptotic responses within pulmonary tissue in a COPD mouse model.

### 2.12. Kaempferol Suppresses Inflammation, Reduces Oxidative Damage, and Inhibits Apoptosis in CSE-Induced BEAS-2B Cells

**Figure 6:**
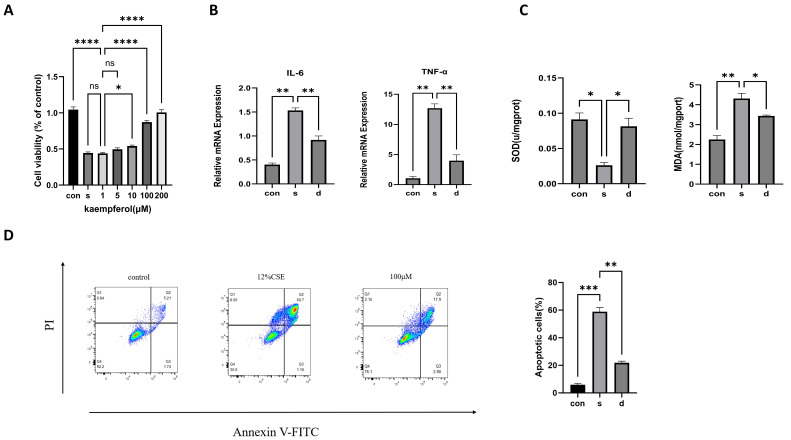
Kaempferol regulates inflammation, oxidative damage, and apoptosis in BEAS-2B cells. (**A**) CCK-8 assay illustrated the viability of cells treated with CSE and different doses of kaempferol. (**B**) RT-qPCR was used to detect IL-6 and TNF-α. (**C**) Contents of SOD and MDA in each group. (**D**) IHC was used to determine the apoptosis rate. * *p* < 0.05, ** *p* < 0.01, *** *p* < 0.001, **** *p* < 0.0001.

BEAS-2B cells were stimulated with CSE to recapitulate the pathological changes of the bronchial epithelium in COPD. CCK-8 analysis was performed to measure cell viability in CSE-induced BEAS-2B cells treated with kaempferol (Figure 6A). After seeding BEAS-2B cells into 96-well plates, the cells were treated with 12% CSE and different doses of kaempferol. Statistical analysis revealed that, versus stimulation with 12% CSE alone, both 100 μM and 200 μM kaempferol significantly improved cell survival rate; 100 μM kaempferol was employed for subsequent experimental analyses in this study. Upon CSE stimulation, the transcript abundance of IL-6 and TNF-α within cultured cells was significantly upregulated, demonstrating that CSE is capable of triggering inflammatory cytokine synthesis (Figure 6B). Treatment with kaempferol markedly blocked the transcript levels of IL-6 and TNF-α, demonstrating its anti-inflammatory activity. After CSE stimulation, the cellular expression level of SOD in cells was decreased, while the expression level of MDA was increased. Upon kaempferol treatment, the expression level of SOD was upregulated, and the concentration of MDA was diminished (Figure 6C). To verify the anti-apoptotic effect of kaempferol, flow cytometric analysis was performed on BEAS-2B cells following Annexin V-FITC/PI staining. A significant degree of apoptosis was observed in BEAS-2B cells after CSE treatment, while kaempferol intervention significantly reduced the proportion of apoptosis in BEAS-2B cells (Figure 6D). Taken together, kaempferol can counteract inflammation and oxidative damage, and apoptosis in CSE-induced BEAS-2B cells.

### 2.13. Transcriptomic Analysis of CSE-Induced BEAS-2B Cells with Kaempferol Treatment

**Figure 7:**
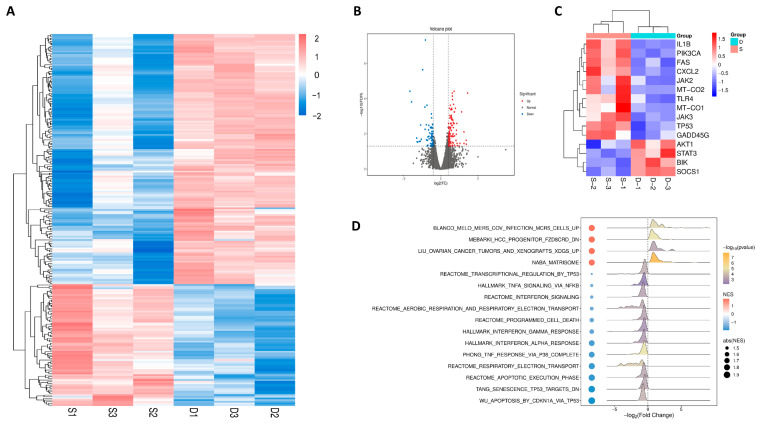
Transcriptome sequencing elucidating the cytoprotective effects of kaempferol on the STAT3-TP53-IL1B signaling network in BASE-2B cells. (**A**) Heatmap of gene expression variations across S and D samples. (**B**) Volcano diagram of differential genes. (**C**) Heatmap of transcription levels of core genes associated with STAT3, TP53, and IL1B. (**D**) GSEA analysis of signaling network-related pathways.

Transcriptome sequencing technology was used to analyze CSE-induced BEAS-2B cells. To investigate whether differential gene expression was significant at the genetic level, a volcano plot of survival-related differential genes in CSE-induced BEAS-2B cells treated with kaempferol was generated (Figure 7B), which intuitively showed the expression variations of associated genes. Through detailed comparison in the model group versus the kaempferol treatment group, a total of 223 genes showed significant changes, among which 73 genes showed reduced expression and 150 genes displayed elevated expression in the treatment group. For the cluster analysis of differential gene expression related to the survival rate of CSE-induced BEAS-2B cells treated with kaempferol, overall, the heatmap exhibited obvious block characteristics, indicating significant differences in gene expression patterns among samples (Figure 7A). Subsequently, cluster analysis was performed on the transcriptional levels of core genes related to the STAT3-TP53-IL1B signaling network (Figure 7C). Versus the model group, the transcriptional expression of IL1B and TP53 in the treatment group was significantly decreased, while the transcriptional levels of STAT3 were significantly increased, and the transcriptional levels of their downstream genes were correspondingly affected. The results of Gene Set Enrichment Analysis (GSEA) uncovered that after kaempferol treatment in the model group, the inflammatory, oxidative damage, and programmed cell death pathways were correspondingly improved (Figure 7D). In essence, kaempferol may exert multiple effects on BASE-2B cells, such as regulating inflammatory responses, oxidative damage, and cellular apoptosis, by modulating the transcription levels of multiple genes in the STAT3-TP53-IL1B signaling network.

## 3. Discussion

This study identified kaempferol as the major therapeutic compound of YFN and recognized three core targets through network pharmacology analysis. In addition, molecular docking and MD simulation were utilized to assess complex stability involving kaempferol and the three key target proteins. Immune infiltration analysis was conducted to further elucidate the immunoregulatory properties of kaempferol against COPD. Subsequently, the COPD mice and CSE-stimulated BEAS-2B cells, combined with transcriptomic analysis, were adopted to assess the ameliorative effects of kaempferol on COPD and investigate its underlying mechanisms.

COPD, a respiratory disease that causes severe harm to human health, has a mainstream management strategy that is primarily pharmacological, covering bronchodilators, inhaled corticosteroids, anti-inflammatory agents, and antibiotics [6]. Mounting clinical evidence indicates that Traditional Chinese Medicine (TCM) exerts a reliable therapeutic effect on COPD, with symptomatic relief, reduced recurrence, improved clinical outcomes, and better quality of life for patients [19,20].

By constructing the component–target network, the core bioactive compounds of YFN, namely quercetin, beta-sitosterol, and kaempferol, were identified. Kaempferol is a natural flavonoid with inhibitory effects on oxidative damage and inflammatory activation, and relevant research has demonstrated that it possesses the ability to attenuate epithelial-mesenchymal transition in bronchial epithelial cells [21,22,23]. Although kaempferol and quercetin exhibit similar biological activities, kaempferol demonstrates stronger inhibitory effects on certain pro-inflammatory markers than quercetin and is more likely to exert its effects in lung tissue [24]. In addition, the low bioavailability and the lack of high-quality randomized controlled trials with lung function indices as endpoints make it difficult for beta-sitosterol to be incorporated into the standardized treatment or research system of COPD [25]. Therefore, we selected kaempferol as the main component for the treatment of COPD in our YFN for in-depth research.

In this study, STAT3, TP53, and IL1B were identified as three key target genes related to COPD. As a key cytosolic transcription factor, STAT3 modulates multiple fundamental cellular functions, such as cell proliferation, survival, and inflammatory responses, acting as a critical inflammatory modulator during COPD progression [26,27,28,29]. TP53 is a critical tumor suppressor gene that participates in multiple processes, encompassing tumor formation, cell cycle control, DNA repair, and apoptosis [30,31,32]. Research has verified that the p53 expression increases significantly in COPD patients, indicating that p53 could serve as an important therapeutic target for cigarette smoke-induced COPD [33]. Interleukin 1 beta, a potent pro-inflammatory released from innate immune cells, is considered a key biomarker associated with the COPD risk [34,35,36]. An increase in the IL1B expression level was also observed in neutrophils from COPD patients [37]. Consequently, STAT3, TP53, and IL1B are most likely to be the therapeutic targets for YFN in COPD treatment.

Kaempferol can inhibit AKT phosphorylation, thereby interfering with PI3K/Akt-mediated inflammatory cascades and cell survival signals, which indirectly suppresses aberrant STAT3 activation [38]. Additionally, kaempferol directly inhibits the STAT3 pathway, suppressing the abundance of pro-inflammatory cytokines, including IL1B, and mitigating chronic airway inflammation and epithelial-mesenchymal transition [39]. In various inflammatory models, kaempferol has been shown to suppress STAT3 activation and nuclear translocation, consequently downregulating STAT3-mediated inflammatory gene expression [40]. Moreover, it significantly reduces IL-1β levels and inhibits the amplification of downstream inflammatory signaling, an anti-inflammatory effect observed in multiple cellular and animal models [41]. Although studies on the direct interaction between TP53 and kaempferol remain limited, evidence suggests that kaempferol can influence p53-mediated apoptosis and cell fate decisions by modulating PI3K/Akt signaling and oxidative damage environments, effects reported in diverse cellular stress models [42]. Therefore, we hypothesize that kaempferol can regulate the STAT3-TP53-IL1B signaling network through multi-target binding, exerting anti-inflammatory, antioxidant, and anti-apoptotic effects to ameliorate COPD.

Furthermore, molecular docking was conducted, revealing hydrogen bonding interactions between kaempferol and amino acid side chains in STAT3, TP53, and IL1B. The MD simulation results suggest that the protein–ligand complexes exhibited high conformational stability, indicating that kaempferol might contribute to the therapeutic effects by interacting with the STAT3-TP53-IL1B signaling network.

The abnormal immune response is considered a critical driver of COPD pathogenesis, and it has been proven that multiple kinds of immune cells participate in the regulation of lung injury and regeneration in COPD, including neutrophils, macrophages, T and B cells [43]. Through the CIBERSORT algorithm, a marked difference in immune cells between COPD and healthy samples was observed. The 22 types of immune cells were found to be closely associated with STAT3, TP53, and IL1B. Therefore, YFN may exert its therapeutic effect mainly through kaempferol, attenuating inflammatory cell infiltration, thereby acting as a crucial modulator of the immune microenvironment in COPD.

In an in vivo study, mice exposed to cigarette smoke (CS) were orally administered kaempferol daily at a dose of 50 mg·kg^−1^·d^−1^ or 100 mg·kg^−1^·d^−1^. In an in vitro experiment, kaempferol was applied at a concentration of 100 μM. The dosage and concentration were determined based on preliminary experimental results evaluating the efficacy and safety of kaempferol. Consistent with these findings, the higher dose significantly increased FEV1/FVC (%) and dynamic lung compliance (Cdyn), while reducing airway resistance (RI), indicating effective alleviation of CS-induced airway obstruction. Histopathological analysis revealed that kaempferol markedly reduced collagen fiber deposition and alleviated collagen deposition around airways compared with the model group. Furthermore, kaempferol treatment significantly attenuated inflammatory cell infiltration and decreased the abundance of proinflammatory mediators, including IL-6, IL-1β, and TNF-α in lung tissue, in addition to IL-6 and TNF-α in BEAS-2B cells, indicating its potent anti-inflammatory activity. Malondialdehyde (MDA) is regarded as a marker of lipid peroxidation, while superoxide dismutase (SOD) represents an important component of the antioxidant protective system [44]. The increased SOD activity and decreased MDA content were observed in both lung tissues and CSE-induced BEAS-2B cells, indicating significant antioxidant effects of kaempferol in COPD. Apoptosis assessment through cleaved caspase-3 immunohistochemistry and Annexin V-FITC/PI staining showed reduced apoptosis in lung tissues and BEAS-2B cells, confirming the anti-apoptotic potential of kaempferol. Transcriptomic analysis further revealed that kaempferol treatment significantly modulated the expression of STAT3, IL1B, TP53, and their downstream genes, suggesting the activation of a multi-target regulatory network. Gene set enrichment analysis (GSEA) indicated that inflammatory, oxidative damage, and programmed cell death pathways were correspondingly improved following kaempferol treatment.

However, this study still has certain limitations. YFN contains a wide array of small-molecular bioactive substances, and this study can only evaluate one of them. Moreover, in this study, only the top three targets with higher ranking scores were selected for the action targets of kaempferol, and no further exploration and analysis were conducted on more potential targets of kaempferol. For the assessment of inflammatory status, analyses were restricted to mouse lung tissues. In cell experiments, only the mRNA levels of two cytokines were measured. Furthermore, regarding the CIBERSORT results, since direct experimental verification was not performed in this study, the findings can only serve as a basis for proposing research hypotheses.

## 4. Materials and Methods

### 4.1. Network Pharmacology

#### 4.1.1. YFN Active Components Screening and Potential Targets Identification

The bioactive components and potential targets of YFN were collected using the Traditional Chinese Medicine Systems Pharmacology Database (TCMSP) (https://www.tcmsp-e.com/tcmsp.php/ (accessed on 20 February 2025)) [45] with the criteria of oral bioavailability (OB) ≥ 30% and drug-likeness (DL) ≥ 0.18 [46,47]. Target proteins were mapped to their unique gene names via the UniProt database (https://www.UniProt.org/ (accessed on 21 February 2025)) by applying the “Reviewed” and “Human” filters [48]. The Excel 2021 was utilized to organize the screened bioactive ingredients and their corresponding genes.

#### 4.1.2. Compound–Target Network Construction

The compound–target network was built and visualized employing Cytoscape software (version 3.10.0) [49]. The bioactive compounds of YFN and their corresponding targets were loaded into Cytoscape 3.10.0, and a network of YFN components and targets was built. In this network, each node represents a component or target, and edges represent the connections between nodes.

#### 4.1.3. Prediction of COPD Targets

The disease targets were screened from resources listed below: GeneCards (https://www.genecards.org/ (accessed on 22 February 2025)) [50], Therapeutic Target Database (https://db.idrblab.net/ttd/ (accessed on 22 February 2025)) [51], Online Mendelian Inheritance in Man (OMIM, http://www.omim.org (accessed on 22 February 2025)) [52] and DrugBank (https://go.drugbank.com/ (accessed on 22 February 2025)) [53]. The keywords “chronic obstructive pulmonary disease” were used for searching. Integrate and deduplicate the retrieval targets to obtain the collection of targets related to COPD.

#### 4.1.4. Construction of Protein–Protein Interaction Network

The Venn diagram tool 2.1.0 (https://bioinfogp.cnb.csic.es/tools/venny/index.html (accessed on 25 February 2025)) [54] was used to identify the overlapping targets between YFN and COPD. Then, the potential targets YFN for the treatment of COPD were imported into the STRING database (http://string-db.org/ (accessed on 25 February 2025)) [55] for protein–protein interaction (PPI) analysis. The species “Homo sapiens” was selected as the filter condition, and the isolated protein was masked. Import PPI information into Cytoscape 3.10.0 for visualization, and the Centiscape2.2 plugin [56] was used. Core targets were selected based on degree, betweenness centrality, and closeness centrality.

#### 4.1.5. GO Enrichment and KEGG Pathway Analysis

We performed Gene Ontology (GO) and Kyoto Encyclopedia of Genes and Genomes (KEGG) enrichment analyses of the overlapping targets via the DAVID database (https://davidbioinformatics.nih.gov/ (accessed on 1 March 2025)) [57]. The top-enriched entries from the biological process (BP), molecular function (MF), cellular component (CC), and KEGG pathways were filtered and graphically represented through an online platform (http://www.bioinformatics.com.cn/ (accessed on 1 March 2025)) [58].

### 4.2. Molecular Docking

The main bioactive constituents were retrieved in mol2 format from TCMSP. Concurrently, the 3D structures of three primary targets were acquired from the Protein Data Bank (PDB, https://www.rcsb.org/ (accessed on 7 March 2025)) [59]. The PyMol 2.6 was employed to eliminate water molecules and original ligands [60]. The selected receptors and the ligands were input into AutoDockTools 1.5.6 for charge calculation processes, and then saved as a pdbqt format [61]. Afterward, we created the grid box, which encompasses the entire receptor protein, and saved the parameters in txt format. Subsequently, molecular docking was conducted through AutoDock Vina Version 1.1.2 (https://vina.scripps.edu (accessed on 7 March 2025)) [62] and visualization through Pymol software and LigPlot+ version 2.2.9 [63].

### 4.3. MD Simulation

To explore the detailed conformational behavior and the robustness of the docked complexes, we chose the Desmond section of Schrödinger to perform MD simulation studies (Release 2022-1, Schrödinger, Inc., New York, NY, USA). The SPC solvent model was selected for the construction of the system, and the salt particles were added according to specific conditions to neutralize the solution system. After that, 0.15 M Na^+^/Cl^−^ was supplemented to mimic the physiological ion concentration. To reduce the adverse atomic clashes, the energy minimization was then carried out. Following that, the MD simulation was conducted, and the results of each complex were shown in different diagrams, including the protein–ligand RMSD, the protein RMSF, the histogram, and the timeline of the protein–ligand contacts [64,65,66].

### 4.4. Data Acquisition and Identification of DEGs in COPD Patients

Datasets GSE130928 and GSE8608 were acquired from the Gene Expression Omnibus (GEO, https://www.ncbi.nlm.nih.gov/geo/ (accessed on 23 July 2025)) [67]. We employed the “limma” R package (version 3.64.1) to conduct differential expression analysis, with thresholds of |logFC| > 1 and an adjusted *p*-value < 0.05. The resulting DEGs were graphically represented using volcano plots and heatmaps [68].

### 4.5. Immune-Infiltrating Landscape of COPD

We applied the CIBERSORT analysis to investigate the immune infiltrating landscape within the microenvironment of patients and healthy people [69]. Subsequently, the analysis of Spearman correlation revealed the association of the immune cell abundance with the expression of the top three core target genes [70]. This integrative approach helped elucidate the correlations between candidate therapeutic targets and particular cell types, clarifying their collective role in COPD pathogenesis.

### 4.6. Materials and Reagents

Kaempferol was supplied by MCE Inc. (Shanghai, China), H-DMEM medium was purchased from Gibco Inc. (Carlsbad, CA, USA), fetal bovine serum was provided by Clark Bioscience Inc. (Hampton, VA, USA), trypsin was obtained from Shanghai Macklin Biochemical Technology Co., Ltd. (Shanghai, China), Trizol reagent was purchased from Thermo Fisher Scientific Inc. (Waltham, MA, USA), chloroform, isopropanol and absolute ethanol were all supplied by Beijing Chemical Works (Beijing, China), dimethyl sulfoxide (DMSO) was acquired from Xilong Chemical Co., Ltd. (Guangzhou, China), RNase-free water was provided by Beijing Solarbio Science & Technology Co., Ltd. (Beijing, China), Annexin V-FITC apoptosis detection kit and total SOD activity assay kit were both obtained from Beyotime Biotechnology Co., Ltd. (Shanghai, China), micro malondialdehyde assay kit was purchased from Nanjing Jiancheng Bioengineering Institute (Nanjing, China), Zunyi cigarette was supplied by Zunyi Cigarette Factory, China Tobacco Guizhou Industrial Co., Ltd. (Zunyi, China), dexamethasone acetate tablets were provided by Zhejiang Xianju Pharmaceutical Co., Ltd. (Taizhou, China), 4% paraformaldehyde solution was purchased from Shanghai Shangbao Biotechnology Co., Ltd. (Shanghai, China), mouse tumor necrosis factor-α (TNF-α) ELISA kit, mouse interleukin-1β (IL-1β) ELISA reagent and mouse interleukin-6 (IL-6) ELISA kit were all acquired from Hubei Pumei Biotechnology Co., Ltd. (Wuhan, China), H&E staining solution and Masson staining solution were both supplied by Wuhan Sevier Biotechnology Co., Ltd. (Wuhan, China), sterile PBS was provided by Euroimmun (Beijing, China), and RNAeasy™ Animal RNA Extraction Kit (Spin Column) was purchased from Beyotime Biotechnology Co., Ltd.

### 4.7. Animals

A total of 60 SPF-grade female Balb/c mice weighing 18 to 22 g were obtained from 536 the Changchun Yisi Experimental Animal Technology Co., Ltd. (Changchun, China). The mice were acclimatized for 7 days under standard housing conditions with a 12-h light/dark cycle and free access to food and water. The 60 female Balb/c mice were randomly divided into 5 experimental groups (*n* = 12 per group). Random numbers were generated using the standard = RAND() function in Microsoft Excel, and the specific grouping was set as follows: (1) normal control group (Con); (2) cigarette smoke-induced COPD model group (Smoke, abbreviated as S); (3) low-dose kaempferol treatment group (DL, administered at 50 mg·kg^−1^·d^−1^); (4) high-dose kaempferol treatment group (DH, administered at 100 mg·kg^−1^·d^−1^); and (5) positive drug treatment group (Y, treated with dexamethasone). A COPD animal model was established via passive cigarette smoke exposure, with cigarettes of the identical brand and batch employed. Except for the control group, all enrolled mice were placed in a transparent airtight plastic chamber measuring 90 × 70 × 40 cm. To ensure normal ventilation and prevent suffocation, a small hole with a radius of 0.2 cm was drilled on each side of the chamber. An air pump was connected to the top hole of the chamber; after cigarettes were lit, manual pressing of the pump delivered smoke at a constant rate into the chamber to form a stable smoke environment for observation and research. The detailed modeling procedure was as follows: mice were exposed to smoke from 6 cigarettes per session, with each session lasting 30 min, twice daily at an interval of 6 h. Smoke exposure was conducted 5 days per week for a total of 20 consecutive weeks. Starting from the 16th week, mice in the DL, DH, and Y groups were intragastrically administered low-dose kaempferol, high-dose kaempferol, and dexamethasone, respectively, while mice in the Control group and model group were given the same volume of normal saline (10 mg/kg) by oral gavage once daily.

### 4.8. Cell Culture and Treatment

The BEAS-2B cell line was obtained from the Cell Bank of the College of Basic Medical 560 Sciences in Jilin University, China. BEAS-2B cells were maintained in DMEM medium containing 10% fetal bovine serum and 1% penicillin-streptomycin. The cells were then maintained in a constant-temperature incubator under controlled conditions of 37 °C and 5% carbon dioxide. BEAS-2B cells were administered with CSE or kaempferol at concentrations of 1, 5, 10, 100, and 200 μM for 24 h.

### 4.9. Cigarette Smoke Extract (CSE) Preparation

Cigarette smoke extract (CSE) was prepared by continuously drawing smoke from unfiltered lit cigarettes via a negative pressure aspirator, with the smoke collected in a sterile vessel. Smoke generated from two cigarettes was bubbled through 10 mL of cell culture medium, followed by filtration for sterilization. The prepared mixture was defined as the CSE stock solution at 100% strength.

### 4.10. Cell Counting Kit-8 (CCK-8) Assay

BEAS-2B cells were seeded into 96-well plates at a density of 5000 cells per well. After cell adhesion, complete medium containing 12% CSE and various concentrations of kaempferol was added to each well, followed by incubation at 37 °C for 24 h. Subsequently, 10% CCK-8 solution was added to each well, and the 96-well plates were further incubated at 37 °C for 1 h. Finally, the absorbance at 450 nm was measured using a microplate reader, and the cell viability was calculated accordingly.

### 4.11. Enzyme-Linked Immunosorbent Assay (ELISA)

The expression levels of IL-6, IL-1β, and TNF-α in mouse lung tissues were detected by ELISA using commercial kits obtained from Hubei Pumei Biotechnology Co., Ltd. (Wuhan, China).

### 4.12. Pathological Staining of Lung Tissue

Lung tissues were perfused with phosphate-buffered saline (PBS) through the right ventricle to remove residual blood. The left lung lobes were then excised, fixed in 4% paraformaldehyde, and embedded in paraffin. Following deparaffinization, 4-μm-thick paraffin sections were stained with hematoxylin and eosin (HE) and Masson’s trichrome.

### 4.13. Real-Time Quantitative Polymerase Chain Reaction (RT-qPCR)

Total cellular RNA was isolated from BEAS-2B cells via TRIzol reagent (Thermo Fisher, Waltham, MA, USA). RT-qPCR was performed on an Applied Biosystems (Foster City, CA, USA) real-time PCR instrument to quantitatively analyze the transcript abundance of IL-6 and TNF-α, respectively. Total RNA from mouse lung tissues was extracted using the RNAeasy™ Animal RNA Extraction Kit (Beyotime, Shanghai, China). RT- PCR was performed on the same RT-qPCR system to quantify the mRNA expression of IL-6, IL-1β, and TNF-α, respectively. The mRNA relative expression levels were determined using the 2^−ΔΔCt^ approach.

### 4.14. Immunohistochemistry (IHC)

Following deparaffinization and rehydration, paraffin-embedded pulmonary sections underwent antigen retrieval for immunohistochemical analysis. Endogenous peroxidase was inactivated by incubating sections in 3% hydrogen peroxide for 25 min. After blocking non-specific binding sites with 10% goat serum for 30 min at room temperature, the sections were incubated overnight at 4 °C with a primary antibody against Cleaved-caspase3. Subsequently, sections were washed and incubated with the corresponding secondary antibody for 1 h at room temperature, counterstained with hematoxylin, and visualized under a light microscope.

### 4.15. Flow Cytometry Assay

Cell status and density were monitored under a microscope. When cell confluence reached 70–80%, cell suspensions were collected. BEAS-2B cells were seeded into 6-well plates at a density of 1 × 10^5^ cells/mL and cultured further in a constant-temperature incubator. Following cell attachment, the cultures were exposed to kaempferol and 12% CSE, followed by incubation for an additional 24 h. The 6-well plates were then collected for subsequent experiments. During the assay, the cell mixture was transferred to a tube designated for centrifugation and spun at 1000× *g* for a duration of 5 min to pellet cellular material. Upon completion of centrifugation, the supernatant was removed, the cellular sediment was resuspended in PBS solution, and the cell precipitate was retained for later use. A total of 195 µL of Annexin V-FITC binding buffer was added to gently resuspend the cells, followed by 5 µL of Annexin V-FITC with thorough mixing. The cells were incubated at room temperature (20–25 °C) in the dark for 10–20 min and then placed in an ice bath. Immediately before detection, 10 µL of propidium iodide (PI) staining solution was added and mixed gently. After preparation, flow cytometric analysis was performed.

### 4.16. Pulmonary Function Testing

The measured parameters included FEV1/FVC%, dynamic lung compliance (Cdyn), and airway resistance (RI). The pulmonary function instrument was calibrated in strict accordance with the operating instructions. The body weight of each mouse was recorded, and mice were anesthetized via intraperitoneal injection of 0.3% pentobarbital sodium solution at a dose of 400 mg/kg. After complete anesthesia, mice were fixed in the supine position. The neck skin was incised, and the surrounding tissues and fascia were separated to fully expose the trachea. A small incision was made on the cartilaginous ring of the trachea for intubation. Following tracheal intubation, the cannula was secured with surgical sutures, and mice were carefully transferred to the detection chamber and connected to the pulmonary function instrument for subsequent testing and data recording. At least six sets of valid data were collected for each parameter.

### 4.17. Assessment of Oxidative Damage

Lung tissues were fully homogenized and centrifuged to obtain the supernatant. Malondialdehyde (MDA) content and superoxide dismutase (SOD) activity in the supernatant were determined using corresponding commercial assay kits, with all procedures performed strictly in accordance with the manufacturer’s instructions. BEAS-2B cell samples were prepared, and MDA and SOD levels were detected using the same method as described above.

### 4.18. Transcriptome Sequencing Technology

Cell suspensions were prepared when cell confluence reached 70–80% and seeded into culture plates. BEAS-2B cells were assigned to three cohorts: a control (Con), a CSE-induced model (S), and a CSE plus kaempferol intervention group (D). After treatment with kaempferol for 24 h, cells were promptly collected on ice and lysed in TRIzol reagent for total RNA extraction. Each experiment was performed in strict biological triplicate, and all procedures were carried out on ice to ensure the integrity and stability of RNA. The extracted samples were then sent to Biomarker Technologies Co., Ltd. (Beijing, China) for subsequent experimental procedures. Raw sequencing reads were filtered using appropriate criteria to remove low-quality reads, reads containing adapter contamination, and reads with a high proportion of unknown N bases.

### 4.19. Statistical Analysis

Statistical assessments were carried out by GraphPad Prism 9 software. Unless otherwise stated, results are expressed as mean ± standard deviation (mean ± SD). Statistical significance was evaluated by Student’s *t*-test for single comparisons. For comparisons among three or more groups, statistical significance was determined by one-way analysis of variance (ANOVA) followed by the Newman–Keuls post hoc test. A value of *p* < 0.05 was considered statistically significant.

## 5. Conclusions

This study first demonstrated that kaempferol exerts therapeutic effects on COPD by inhibiting inflammatory responses, oxidative damage, and cell apoptosis. Additionally, transcriptomics and network pharmacology results indicated that the therapeutic mechanism of YFN in COPD potentially involves targeting the STAT3-TP53-IL1B signaling network via kaempferol.

## Figures and Tables

**Table 1:** The shared bioactive constituents among the ingredients.

MOL ID	Molecule Name	OB	DL	Origin *	Name
MOL007514	methyl icosa-11,14-dienoate	39.67	0.23	DS, FF	A1
MOL002879	Diop	43.59	0.39	DS, SZY, BJT	A2
MOL000449	Stigmasterol	43.83	0.76	DS, SZY, FBX	A3
MOL004355	Spinasterol	42.98	0.76	DS, GL	A4
MOL000006	luteolin	36.16	0.25	DS, DANS	A5
MOL007059	3-beta-Hydroxymethyllenetanshiquinone	32.16	0.41	DS, DANS	A6
MOL000033	(3S,8S,9S,10R,13R,14S,17R)-10,13-dimethyl-17-[(2R,5S)-5-propan-2-yloctan-2-yl]-2,3,4,7,8,9,11,12,14,15,16,17-dodecahydro-1H-cyclopenta[a]phenanthren-3-ol	36.23	0.78	HQ, BQ	B1
MOL000354	isorhamnetin	49.6	0.31	HQ, TSZ	B2
MOL000422	kaempferol	41.88	0.24	HQ, TSZ	B3
MOL000098	quercetin	46.43	0.28	HQ, TSZ	B4
MOL000296	hederagenin	36.91	0.75	HQ, TR	B5
MOL000358	beta-sitosterol	36.91	0.75	FF, HJ, SZY, TSZ, BJT, FBX, ZBM, TR	C1
MOL000359	sitosterol	36.91	0.75	FF, HJ, SZY, BJT	C2
MOL001494	Mandenol	42	0.19	FF, SZY, GL	C3
MOL001942	isoimperatorin	45.46	0.23	FF, DS	C4
MOL002714	baicalein	33.52	0.21	HJ, FBX	D1
MOL002883	Ethyl oleate (NF)	32.4	0.19	SZY, BJT	E1
MOL005530	Hydroxygenkwanin	36.47	0.27	SZY, GL	E2
MOL001771	poriferast-5-en-3beta-ol	36.91	0.75	SZY, DS	E3
MOL002776	Baicalin	40.12	0.75	FBX, DS	F1

* Abbreviations: DS, Radix Codonopsis; FF, Radix Ledebouriellae; SZY, Fructus Corni; HJ, Rhizoma Polygonati; TSZ, Semen Cuscutae; BJT, Radix Morindae Officinalis; GL, Fructus Trichosanthis; FBX, Rhizoma Pinelliae; ZBM, Bulbus Fritillariae Thunbergii; DANS, Radix Salviae Miltiorrhizae; TR, Semen Persicae; HQ, Radix Astragali.

**Table 2:** Top three bioactive compounds information of YFN network.

Compound	Molecule Structure	Degree	Average Shortest PathLength	Betweenness Centrality	Closeness Centrality
quercetin	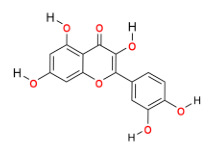	302	2.088709677	0.322894415	0.478764479
beta-sitosterol	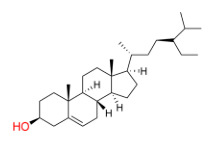	296	2.512096774	0.035293707	0.398073836
kaempferol	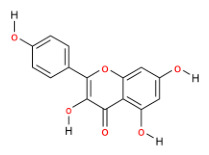	130	2.411290323	0.069965394	0.414715719

**Table 3:** Binding affinity (kcal/mol) between kaempferol and the key targets.

Target	Target (PDBID)	Target Structure	Affinity (kcal/mol)
STAT3	6TLC	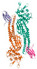	−7.686
TP53	7DVD	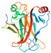	−7.814
IL1B	7Z4T	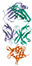	−7.306

## Data Availability

All data are contained in the article.

## Abbreviations

The following abbreviations are used in this manuscript:

YFN	Yufeining
COPD	Chronic obstructive pulmonary disease
DS	Radix Codonopsis
HQ	Radix Astragali
BZ	Rhizoma Atractylodis Macrocephalae
FF	Radix Ledebouriellae
HJ	Rhizoma Polygonati
SZY	Fructus Corni
WWZ	Fructus Schisandrae
HTR	Juglandis
TSZ	Semen Cuscutae
BJT	Radix Morindae Officinalis
GL	Fructus Trichosanthis
FBX	Rhizoma Pinelliae
ZBM	Bulbus Fritillariae Thunbergii
DANS	Radix Salviae Miltiorrhizae
TR	Semen Persicae
TCMSP	Traditional Chinese Medicine Systems Pharmacology Database
DL	Drug-Likeness
OB	Oral bioavailability
GEO	Gene Expression Omnibus
OMIM	Online Mendelian Inheritance in Man
PPI	Protein–protein interaction
BP	Biological process
CC	Cellular component
MF	Molecular function
GO	Gene Ontology
KEGG	Kyoto Encyclopedia of Genes and Genomes
PDB	Protein Data Bank
STAT3	Signal transducer and activator of transcription 3
IL1B	Interleukin 1 beta
DEGs	Differentially expressed genes
CS	Cigarette smoke
CSE	Cigarette smoke extract
CCK-8	Cell counting kit-8
ELISA	Enzyme-linked immunosorbent assay
HE	Hematoxylin and eosin
MASSON	Masson’s trichrome
RT-qPCR	Real-time quantitative polymerase chain reaction
IHC	Immunohistochemistry
Cdyn	Dynamic lung compliance
RI	Airway resistance
SOD	Superoxide Dismutase
MDA	Malondialdehyde
GSEA	Gene set enrichment analysis

## References

[1] Celli B., Fabbri L., Criner G., Martinez F.J., Mannino D., Vogelmeier C., Montes de Oca M., Papi A., Sin D.D., Han M.K. (2022). Definition and Nomenclature of Chronic Obstructive Pulmonary Disease: Time for Its Revision. Am. J. Respir. Crit. Care Med..

[2] Christenson S.A., Smith B.M., Bafadhel M., Putcha N. (2022). Chronic obstructive pulmonary disease. Lancet.

[3] Greene C.M., Marciniak S.J., Teckman J., Ferrarotti I., Brantly M.L., Lomas D.A., Stoller J.K., McElvaney N.G. (2016). α1-Antitrypsin deficiency. Nat. Rev. Dis. Primers.

[4] Boers E., Barrett M., Su J.G., Benjafield A.V., Sinha S., Kaye L., Zar H.J., Vuong V., Tellez D., Gondalia R. (2023). Global Burden of Chronic Obstructive Pulmonary Disease Through 2050. JAMA Netw. Open.

[5] Mannino D.M., Buist A.S. (2007). Global burden of COPD: Risk factors, prevalence, and future trends. Lancet.

[6] Singh D., Agusti A., Anzueto A., Barnes P.J., Bourbeau J., Celli B.R., Criner G.J., Frith P., Halpin D.M.G., Han M. (2019). Global Strategy for the Diagnosis, Management, and Prevention of Chronic Obstructive Lung Disease: The GOLD science committee report 2019. Eur. Respir. J..

[7] Jones B., Donovan C., Liu G., Gomez H.M., Chimankar V., Harrison C.L., Wiegman C.H., Adcock I.M., Knight D.A., Hirota J.A. (2017). Animal models of COPD: What do they tell us?. Respirology.

[8] Yang K., Wang J., Ma Y., Zhang H. (2026). Advances in Traditional Chinese Medicine for chronic obstructive pulmonary disease through multi-omics approaches. Front. Cell Dev. Biol..

[9] Hong M.L., Yang G.Z., Chen W.X., Gao L.Y., Cai S.H., Dai S.Z. (2005). Effect of Yufeining on induced sputum interleukin-8 in patients with chronic obstructive pulmonary disease at the stable phase. Chin. J. Integr. Med..

[10] Hong M., Hong C., Chen H., Ke G., Huang J., Huang X., Liu Y., Li F., Li C. (2018). Effects of the Chinese herb formula Yufeining on stable chronic obstructive pulmonary disease: A randomized, double-blind, placebo-controlled trial. Medicine.

[11] Shadman J., Haghi-Aminjan H., Alipour M.R., Panahpour H. (2025). The Neuroprotective Mechanisms of Kaempferol in Experimental Ischemic Stroke: A Preclinical Systematic Review. Mol. Neurobiol..

[12] Yao Y., Wan R., An Y., He B., Tan Q., Xu H., Pan X., Liu S., Chen X., Li J. (2026). Kaempferol ameliorates renal injury by inhibiting Piezo1/HIF-1a/ROS/NLRP3 signaling in chronic kidney disease. Biochem. Pharmacol..

[13] Zhang X., Xie Y., Cai Y., Huang H., Liang H., Liao G., Jiang Y., Peng X., Zhan S., Huang X. (2024). RNA-seq analysis and in vivo experiments identified the protective effect of kaempferol on idiopathic pulmonary fibrosis by regulating the PPARG/TNC signaling pathway to reduce ECM deposition. Food Funct..

[14] Shao D., Liu X., Wu J., Zhang A., Bai Y., Zhao P., Li J. (2022). Identification of the active compounds and functional mechanisms of Jinshui Huanxian formula in pulmonary fibrosis by integrating serum pharmacochemistry with network pharmacology. Phytomedicine.

[15] Bisht A., Tewari D., Kumar S., Chandra S. (2023). Network pharmacology, molecular docking, and molecular dynamics simulation to elucidate the mechanism of anti-aging action of Tinospora cordifolia. Mol. Divers..

[16] Nogales C., Mamdouh Z.M., List M., Kiel C., Casas A.I., Schmidt H.H.H.W. (2022). Network pharmacology: Curing causal mechanisms instead of treating symptoms. Trends Pharmacol. Sci..

[17] Wang Z.-y., Li M.-z., Li W.-j., Ouyang J.-f., Gou X.-j., Huang Y. (2023). Mechanism of action of Daqinjiao decoction in treating cerebral small vessel disease explored using network pharmacology and molecular docking technology. Phytomedicine.

[18] Liu X., Cui S., Li W., Xie H., Shi L. (2023). Elucidation of the anti-colon cancer mechanism of Phellinus baumii polyphenol by an integrative approach of network pharmacology and experimental verification. Int. J. Biol. Macromol..

[19] Xiao Q., Meng Y., Wang G., Wang M., Meng Y., Zhou M. (2025). Effects of Wenfei Guyuan umbilical moxibustion on patients with stable chronic obstructive pulmonary disease: A multicenter randomized controlled trial. Complement. Ther. Med..

[20] Jiansheng L., Haifeng W., Suyun L., Hailong Z., Xueqing Y., Xiaoyun Z., Fengsen L., Xianmei Z., Zikai S., Yimin M. (2016). Effect of sequential treatment with TCM syndrome differentiation on acute exacerbation of chronic obstructive pulmonary disease and AECOPD risk window. Complement. Ther. Med..

[21] Hong F., Zhao M., Xue L.-L., Ma X., Liu L., Cai X.-Y., Zhang R.-J., Li N., Wang L., Ni H.-F. (2022). The ethanolic extract of Artemisia anomala exerts anti-inflammatory effects via inhibition of NLRP3 inflammasome. Phytomedicine.

[22] Gong J.-H., Cho I.-H., Shin D., Han S.-Y., Park S.-H., Kang Y.-H. (2014). Inhibition of airway epithelial-to-mesenchymal transition and fibrosis by kaempferol in endotoxin-induced epithelial cells and ovalbumin-sensitized mice. Lab. Investig..

[23] He Y.-Q., Zhou C.-C., Yu L.-Y., Wang L., Deng J.-l., Tao Y.-L., Zhang F., Chen W.-S. (2021). Natural product derived phytochemicals in managing acute lung injury by multiple mechanisms. Pharmacol. Res..

[24] Crespo I., García-Mediavilla M.V., Gutiérrez B., Sánchez-Campos S., Tuñón M.J., González-Gallego J. (2008). A comparison of the effects of kaempferol and quercetin on cytokine-induced pro-inflammatory status of cultured human endothelial cells. Br. J. Nutr..

[25] Bin Sayeed M.S., Karim S.M.R., Sharmin T., Morshed M.M. (2016). Critical Analysis on Characterization, Systemic Effect, and Therapeutic Potential of Beta-Sitosterol: A Plant-Derived Orphan Phytosterol. Medicines.

[26] Zou S., Tong Q., Liu B., Huang W., Tian Y., Fu X. (2020). Targeting STAT3 in Cancer Immunotherapy. Mol. Cancer.

[27] Wang D., Li S., Yang Z., Yu C., Wu P., Yang Y., Zhang R., Li Q., Yang J., Li H. (2024). Single-cell transcriptome analysis deciphers the CD74-mediated immune evasion and tumour growth in lung squamous cell carcinoma with chronic obstructive pulmonary disease. Clin. Transl. Med..

[28] Kiszałkiewicz J.M., Majewski S., Piotrowski W.J., Górski P., Pastuszak-Lewandoska D., Migdalska-Sęk M., Brzeziańska-Lasota E. (2021). Evaluation of selected IL6/STAT3 pathway molecules and miRNA expression in chronic obstructive pulmonary disease. Sci. Rep..

[29] Tang H., Wilson A.C., Rocco A., Chiles J., Srinivasasainagendra V., Labaki W., Meyers D., Hidalgo B., Irvin M.R., Bhatt S.P. (2025). Novel risk loci encompassing genes influencing STAT3, GPCR, and oxidative stress signaling are associated with co-morbid GERD and COPD. PLoS Genet..

[30] Mogi A., Kuwano H., Doetsch P.W. (2011). TP53 Mutations in Nonsmall Cell Lung Cancer. BioMed Res. Int..

[31] Vogelstein B., Lane D., Levine A.J. (2000). Surfing the p53 network. Nature.

[32] Levine A.J. (1997). p53, the cellular gatekeeper for growth and division. Cell.

[33] Gouda M.M., Shaikh S.B., Chengappa D., Kandhal I., Shetty A., Bhandary Y. (2018). Changes in the expression level of IL-17A and p53-fibrinolytic system in smokers with or without COPD. Mol. Biol. Rep..

[34] Lopez-Castejon G., Brough D. (2011). Understanding the mechanism of IL-1β secretion. Cytokine Growth Factor Rev..

[35] Yi G., Liang M., Li M., Fang X., Liu J., Lai Y., Chen J., Yao W., Feng X., Hu L. (2018). A large lung gene expression study identifying IL1B as a novel player in airway inflammation in COPD airway epithelial cells. Inflamm. Res..

[36] Xie Z.-K., Huang Q.-P., Huang J., Xie Z.-F. (2014). Association between the IL1B, IL1RN polymorphisms and COPD risk: A meta-analysis. Sci. Rep..

[37] Mariotti B., Bracaglia C., Gasperini S., Sartori G., Crisafulli E., Bazzoni F. (2025). Innate immune reprogramming in circulating neutrophils of COPD patients. J. Allergy Clin. Immunol..

[38] Kaur S., Mendonca P., Soliman K.F.A. (2024). The Anticancer Effects and Therapeutic Potential of Kaempferol in Triple-Negative Breast Cancer. Nutrients.

[39] Chin H.K., Horng C.T., Liu Y.S., Lu C.C., Su C.Y., Chen P.S., Chiu H.Y., Tsai F.J., Shieh P.C., Yang J.S. (2018). Kaempferol inhibits angiogenic ability by targeting VEGF receptor-2 and downregulating the PI3K/AKT, MEK and ERK pathways in VEGF-stimulated human umbilical vein endothelial cells. Oncol. Rep..

[40] Chen A.Y., Chen Y.C. (2013). A review of the dietary flavonoid, kaempferol on human health and cancer chemoprevention. Food Chem..

[41] Basu A., Das A.S., Sharma M., Pathak M.P., Chattopadhyay P., Biswas K., Mukhopadhyay R. (2017). STAT3 and NF-κB are common targets for kaempferol-mediated attenuation of COX-2 expression in IL-6-induced macrophages and carrageenan-induced mouse paw edema. Biochem. Biophys. Rep..

[42] Alam W., Khan H., Shah M.A., Cauli O., Saso L. (2020). Kaempferol as a Dietary Anti-Inflammatory Agent: Current Therapeutic Standing. Molecules.

[43] Kapellos T.S., Conlon T.M., Yildirim A.Ö., Lehmann M. (2023). The impact of the immune system on lung injury and regeneration in COPD. Eur. Respir. J..

[44] Poljsak B., Jamnik P., Milisav I. (2025). The Importance of Multifaceted Approach for Accurate and Comprehensive Evaluation of Oxidative Stress Status in Biological Systems. Antioxidants.

[45] Ru J., Li P., Wang J., Zhou W., Li B., Huang C., Li P., Guo Z., Tao W., Yang Y. (2014). TCMSP: A database of systems pharmacology for drug discovery from herbal medicines. J. Cheminform..

[46] Li X., Wei S., Niu S., Ma X., Li H., Jing M., Zhao Y. (2022). Network pharmacology prediction and molecular docking-based strategy to explore the potential mechanism of Huanglian Jiedu Decoction against sepsis. Comput. Biol. Med..

[47] Duan Z.L., Wang Y.J., Lu Z.H., Tian L., Xia Z.Q., Wang K.L., Chen T., Wang R., Feng Z.Y., Shi G.P. (2023). Wumei Wan attenuates angiogenesis and inflammation by modulating RAGE signaling pathway in IBD: Network pharmacology analysis and experimental evidence. Phytomedicine.

[48] (2015). UniProt: A hub for protein information. Nucleic Acids Res..

[49] Shannon P., Markiel A., Ozier O., Baliga N.S., Wang J.T., Ramage D., Amin N., Schwikowski B., Ideker T. (2003). Cytoscape: A software environment for integrated models of biomolecular interaction networks. Genome Res..

[50] Rebhan M., Chalifa-Caspi V., Prilusky J., Lancet D. (1997). GeneCards: Integrating information about genes, proteins and diseases. Trends Genet..

[51] Wang Y., Zhang S., Li F., Zhou Y., Zhang Y., Wang Z., Zhang R., Zhu J., Ren Y., Tan Y. (2020). Therapeutic target database 2020: Enriched resource for facilitating research and early development of targeted therapeutics. Nucleic Acids Res..

[52] Amberger J.S., Bocchini C.A., Schiettecatte F., Scott A.F., Hamosh A. (2015). OMIM.org: Online Mendelian Inheritance in Man (OMIM®), an online catalog of human genes and genetic disorders. Nucleic Acids Res..

[53] Knox C., Wilson M., Klinger C.M., Franklin M., Oler E., Wilson A., Pon A., Cox J., Chin N.E.L., Strawbridge S.A. (2024). DrugBank 6.0: The DrugBank Knowledgebase for 2024. Nucleic Acids Res..

[54] Yang Y., Zhou X., Jia G., Li T., Li Y., Zhao R., Wang Y. (2023). Network pharmacology based research into the effect and potential mechanism of Portulaca oleracea L. polysaccharide against ulcerative colitis. Comput. Biol. Med..

[55] von Mering C., Huynen M., Jaeggi D., Schmidt S., Bork P., Snel B. (2003). STRING: A database of predicted functional associations between proteins. Nucleic Acids Res..

[56] Feng L., Sun R., Zhang H., Zhang J., Peng Z., Li J., Gao Y., Xu Y., Cui J., Liu J. (2025). Exploring the protective mechanisms of syringaresinol against myocardial infarction by experimental validation and network pharmacology. Biochim. Biophys. Acta Mol. Basis Dis..

[57] Sherman B.T., Hao M., Qiu J., Jiao X., Baseler M.W., Lane H.C., Imamichi T., Chang W. (2022). DAVID: A web server for functional enrichment analysis and functional annotation of gene lists (2021 update). Nucleic Acids Res..

[58] Tang D., Chen M., Huang X., Zhang G., Zeng L., Zhang G., Wu S., Wang Y. (2023). SRplot: A free online platform for data visualization and graphing. PLoS ONE.

[59] Berman H.M., Westbrook J., Feng Z., Gilliland G., Bhat T.N., Weissig H., Shindyalov I.N., Bourne P.E. (2000). The Protein Data Bank. Nucleic Acids Res..

[60] Lu S., Sun X., Zhou Z., Tang H., Xiao R., Lv Q., Wang B., Qu J., Yu J., Sun F. (2023). Mechanism of Bazhen decoction in the treatment of colorectal cancer based on network pharmacology, molecular docking, and experimental validation. Front. Immunol..

[61] Zhang H., Xiong P., Zheng T., Hu Y., Guo P., Shen T., Zhou X. (2025). Combination of Berberine and Evodiamine Alleviates Obesity by Promoting Browning in 3T3-L1 Cells and High-Fat Diet-Induced Mice. Int. J. Mol. Sci..

[62] Wu Y., Liu X., Li G. (2022). Integrated bioinformatics and network pharmacology to identify the therapeutic target and molecular mechanisms of Huangqin decoction on ulcerative Colitis. Sci. Rep..

[63] Laskowski R.A., Swindells M.B. (2011). LigPlot+: Multiple ligand-protein interaction diagrams for drug discovery. J. Chem. Inf. Model..

[64] Ahamed N.A., Arif I.A. (2023). Finding potential inhibitors for Main protease (Mpro) of SARS-CoV-2 through virtual screening and MD simulation studies. Saudi J. Biol. Sci..

[65] Zhao Z., Wang W., Pang J., Zhou B., Li X., Wang Y., Zheng K., Ren Z. (2025). Computational design and evaluation of multiepitope vaccines against herpes simplex virus type 1. Front. Immunol..

[66] Mandal S.K., Kumar B.K., Sharma P.K., Murugesan S., Deepa P.R. (2022). In silico and in vitro analysis of PPAR—α/γ dual agonists: Comparative evaluation of potential phytochemicals with anti-obesity drug orlistat. Comput. Biol. Med..

[67] Edgar R., Domrachev M., Lash A.E. (2002). Gene Expression Omnibus: NCBI gene expression and hybridization array data repository. Nucleic Acids Res..

[68] Ritchie M.E., Phipson B., Wu D., Hu Y., Law C.W., Shi W., Smyth G.K. (2015). limma powers differential expression analyses for RNA-sequencing and microarray studies. Nucleic Acids Res..

[69] Newman A.M., Liu C.L., Green M.R., Gentles A.J., Feng W., Xu Y., Hoang C.D., Diehn M., Alizadeh A.A. (2015). Robust enumeration of cell subsets from tissue expression profiles. Nat. Methods.

[70] He X., Zhang J., Gong M., Gu Y., Dong B., Pang X., Zhang C., Cui Y. (2023). Identification of potential ferroptosis-associated biomarkers in rheumatoid arthritis. Front. Immunol..

